# High Infection Fatality Rate Among Elderly and Risk Factors Associated With Infection Fatality Rate and Asymptomatic Infections of COVID-19 Cases in Hong Kong

**DOI:** 10.3389/fmed.2021.678347

**Published:** 2021-05-24

**Authors:** Jun Tao, Xiaoyu Zhang, Salihu S. Musa, Lin Yang, Daihai He

**Affiliations:** ^1^School of Public Health, Li Ka Shing Faculty of Medicine, The University of Hong Kong, Hong Kong, China; ^2^Department of Applied Mathematics, Hong Kong Polytechnic University, Hong Kong, China; ^3^School of Nursing, Hong Kong Polytechnic University, Hong Kong, China

**Keywords:** asymptomatic COVID-19 cases, Hong Kong, boosted regression tree, elderly, infection fatality rate

## Abstract

**Background:** Since the emergence in December 2019, the COVID-19 pandemic has become one of the greatest global public health threats in history. However, asymptomatic infections have increased the challenges of providing accurate estimates for the infection fatality rate (IFR) of COVID-19.

**Methods:** We calculated the asymptomatic case ratios based on the reported COVID-19 cases in Hong Kong where intensive testing has been conducted in close contacts and high-risk populations. We estimated the IFR using both symptomatic and asymptomatic cases as denominator. The boosted regression tree (BRT) and multivariable logistic regression models were used to identify relative contribution and effect size of the risk factors associated with the asymptomatic cases and IFRs.

**Results:** The ratio of the asymptomatic patients in Hong Kong was higher than many other regions over the world. Imported cases had a higher asymptomatic proportion than local cases. Older age and male were associated with a higher IFR than younger age and females.

**Conclusion:** Policymakers should consider the potential risk factors for the asymptomatic infections and IFRs by the Hong Kong surveillance data to mitigate the diseases and reduce the case mortality of COVID-19.

## Introduction

Since its emergence in December 2019, the COVID-19 pandemic has become one of the greatest global public health threats in history. As of 8 March 2021, more than 116 million cases have been confirmed, with more than 2.59 million deaths attributed to COVID-19 (1). As the pandemic spread, it has been found that some cases did not manifest any clinical symptoms, which could have been missed by the surveillance system based on healthcare settings, rendering serious underestimation of disease burden (2). Recent studies have reported that 13.1–27.4% of COVID-19 cases were asymptomatic, depending on geographical regions, case definitions and the length of follow-up period (3–6). The lack of reliable data on asymptomatic cases in different age groups hinders accurate estimation of age-specific infection fatality rates (IFRs) for COVID-19, an important indicator for disease burden of the pandemic.

The infection fatality ratio is a critical estimation for the disease burden of the COVID-19 pandemic globally. It is of great policy value to have accurate and up-to-date estimates in different population stratifications so that appropriate prevention measures could be in place to mitigate risk. It is important to note that the infection fatality ratio is not constant among different populations because it depends on the age distribution in the population and the infection rates in each age group, the asymptomatic case ratios, and many other risk factors. There are mainly two approaches to estimate the IFRs, including the seroprevalence and the comprehensive tracing studies (7). Some research adopted the result of serological tests to estimate the cumulative infections among populations considering the limited testing capacity and asymptomatic cases, but the accuracy of serological tests also depends on the humoral immune response, the waning speed of antibodies in the circulation and testing sensitivity (8). Besides, some seroprevalence studies are based on “convenience samples” collected for other purposes and the sampling frame cannot represent the general population (7).

There are two approaches to estimate the IFRs, including the seroprevalence studies testing the antibody to SAR-CoV-2 or comprehensive case tracing by extensive live virus testing (7). The accuracy of IFRs estimated by case tracing is influenced by the extent to which infected individuals are identified, especially the asymptomatic cases (7). The IFRs could vary greatly across the world and depend crucially on the age groups infected in the specific region, with the elderly of significantly higher IFRs (7). Around the globe, Hong Kong is unique in its geographical proximity to mainland China and its preparedness to emerging infectious diseases based on the lessons learnt during SARS and avian influenza epidemics. In addition, the persistent efforts for contact tracing and testing among the local residents have contributed to the relatively robust surveillance system.

The city has experienced four waves of the COVID-19 outbreaks in communities and opened the access to deidentified data of individual COVID-19 cases. The Hong Kong surveillance data can provide good resources to estimate the asymptomatic ratios and IFRs by age group. Herein, the asymptomatic ratios and IFRs were estimated together with their corresponding risk factors to provide critical information for health policymaking.

## Methods

The study used the Hong Kong COVID-19 surveillance data, which is publicly available from the Centre for Health Protection (CHP) in Hong Kong (9). By 27th January 2021, Hong Kong has recorded 10,283 confirmed cases of COVID-19. Among these cases, 2913 were asymptomatic and 174 were fatal cases. The average age of patients was 45.0 ± 19.9 years, ranging from 14 days to 100 years. About half (48.4%) of them were men and over 80% were local residents. The 1,041 cases reported in the first and second wave were combined together for analysis due to the similarity in control measures and the relatively small sample size (95 cases) in the first wave.

The demographic characteristics included age and gender, and the classification variables included the waves of COVID-19 outbreak which individual case was identified in, and classification of local and imported cases. We defined the first wave as the period of January 23rd 2020- February 29th 2020, the second wave as March 1st 2020–May 6th 2020, the third wave as May 7th 2020–November 22nd 2020, and the fourth wave as November 23rd 2020–January 27th 2021 (10). Six categories of cases were identified in the original data, including local/imported case, possibly local case, epidemiologically linked with local/imported case, epidemiologically linked with possibly local case. We classified local, possibly local, epidemiologically linked with possibly local case as local cases and the other three as imported cases. The proportion of asymptomatic cases and case fatality were set as the outcome variables. The distribution of each explanatory variable was firstly examined using Chi-square test for categorical variables and one-way analysis of variance (ANOVA) for continuous variables.

To quantify the relative contributions of these characteristics to the asymptomatic ratios and IFRs, the boosted regression tree (BRT) model was applied using package “gbm Version 2.1.5” in R Version 3.6.2. Three important model parameters were considered in the BRT model, including number of trees, learning rate and tree complexity. We tested values of tree complexity from 1 to 8 in the BRT model, and selected optimal value when further adding one level would not increase the cross-validated area under curve (AUC). The values of 2 and 4 were selected with AUC of 0.711 and 0.934 for outcome 1 and outcome 2, respectively. Learning rate, known as shrinkage, is set as 0.005. A bagging factor of 0.5 was set for boosting in the BRT model, as applied in the previous analysis (11). The multivariable logistic regression model was used to estimate the effect sizes of explanatory variables on each outcome. The adjusted odds ratio (AOR) and their 95% confidence intervals (95% CI) were then presented in the forest plot. The age and gender are reported to be potential influencing factors for asymptomatic COVID-19 infection and its infection fatality rate (12). We also included the case classification in the model since a previous study reported that the proportion of asymptomatic cases was relatively higher in the imported travelers than in local cases (13). This correlation is reasonable because imported cases are selected samples who have gone through temperature testing before travel. Aside from the age, gender and case classification, the dummy variables of four waves were also included in the regression to adjust for temporal change in local healthcare capacity and the dominant viral strains.

The asymptomatic ratio (the number of asymptomatic cases divided by the total cases) was plotted in combination with the case classification and the infection fatality rate (IFR: the number of fatal cases divided by the total cases) was plotted by age, in **Figures 3**, **4**, respectively. **Figure 3** shows the proportion of asymptomatic COVID-19 patients plotted in red dots with a 95% confidence interval indicated by the vertical red lines. The serial trend was simulated in the smooth black curve. The classification was shown in the bars, with the gray representing the local cases and the white representing imported cases. In **Figure 4**, the trends of IFR against age in Hong Kong and a previous report which included a global estimate from ten representative serological studies (14). IFRs by age in Hong Kong are shown in black dots and IFRs in other countries are shown in red squares. The rates were plotted in dots with 95% confidence interval indicated by the vertical lines. We used local cases only for calculation of infection fatality rate in Hong Kong since there are few cases were imported (4 out of 174).

The time series plot of daily confirmed cases was plotted in **Figure 5**. The number of all daily cases was plotted in black dots, and local cases were indicated in red triangles. The number of confirmed cases identified from the universal testing was plotted from September 4th to September 14th, shown in blue dots. The whole universal testing was lasting for 14 days since September 1st, with 42 confirmed cases identified from the testing directly or indirectly, over about 1.78 million participants.

## Results

Table 1 shows the proportion of asymptomatic cases was higher in patients aged 20 years or younger, in imported cases and the third and fourth wave. Meanwhile, the prevalence of deceased cases was higher in men than women, in patients with older ages, and local cases. Among the several waves in Hong Kong, more deceased cases were reported in the third wave.

**Table 1:** Potential explanatory variables of cases according to interested outcomes.

	**Cases without interested outcomes**	**Cases with interested outcomes**	***P*-value^a^**
**Outcome1: Asymptomatic**			
**Age**	46.5 (19.6)	41.3 (20.4)	<0.001
<20 years	607 (58.1)	437 (41.9)	<0.001
20–65 years	5,500 (72.6)	2,075 (27.4)	
>65 years	1,263 (75.9)	401 (24.1)	
**Gender**			0.12
Female	3,842 (72.4)	1,468 (27.6)	
Male	3,528 (70.9)	1,445 (29.1)	
**Wave**			<0.001
1st and 2nd wave	845 (81.2)	196 (18.8)	
3rd wave	3,191 (69.6)	1,397 (30.4)	
4th wave	3,334 (71.6)	1,320 (28.4)	
**Case classification**			<0.001
Local	6,583 (79)	1,754 (21)	
Imported	787 (40.4)	1,159 (59.6)	
**Outcome2: Deceased^b^**			
**Age**	44.4 (19.5)	78.8 (11.2)	<0.001
<20 years	1,035 (100)	0 (0)	<0.001
20–65 years	7,514 (99.7)	21 (0.3)	
>65 years	1,500 (90.7)	153 (9.3)	
**Gender**			0.02
Female	5,206 (98.6)	74 (1.4)	
Male	4,843 (98)	100 (2)	
**Wave**			<0.001
1st and 2nd wave	1,036 (99.5)	5 (0.5)	
3rd wave	4,483 (97.7)	105 (2.3)	
4th wave	4,530 (98.6)	64 (1.4)	
**Case classification**			<0.001
Local	8,110 (97.9)	170 (2.1)	
Imported	1,939 (99.8)	4 (0.2)	

a *P-values were obtained by using one-way analysis of variance (ANOVA) for continuous variable (age), and chi-square test for other categorical variables*.

b *The total number used for the analysis of outcome 2 is 10,223 instead of 10,283 due to the 1-day lag in reporting*.

**Figure 1:**
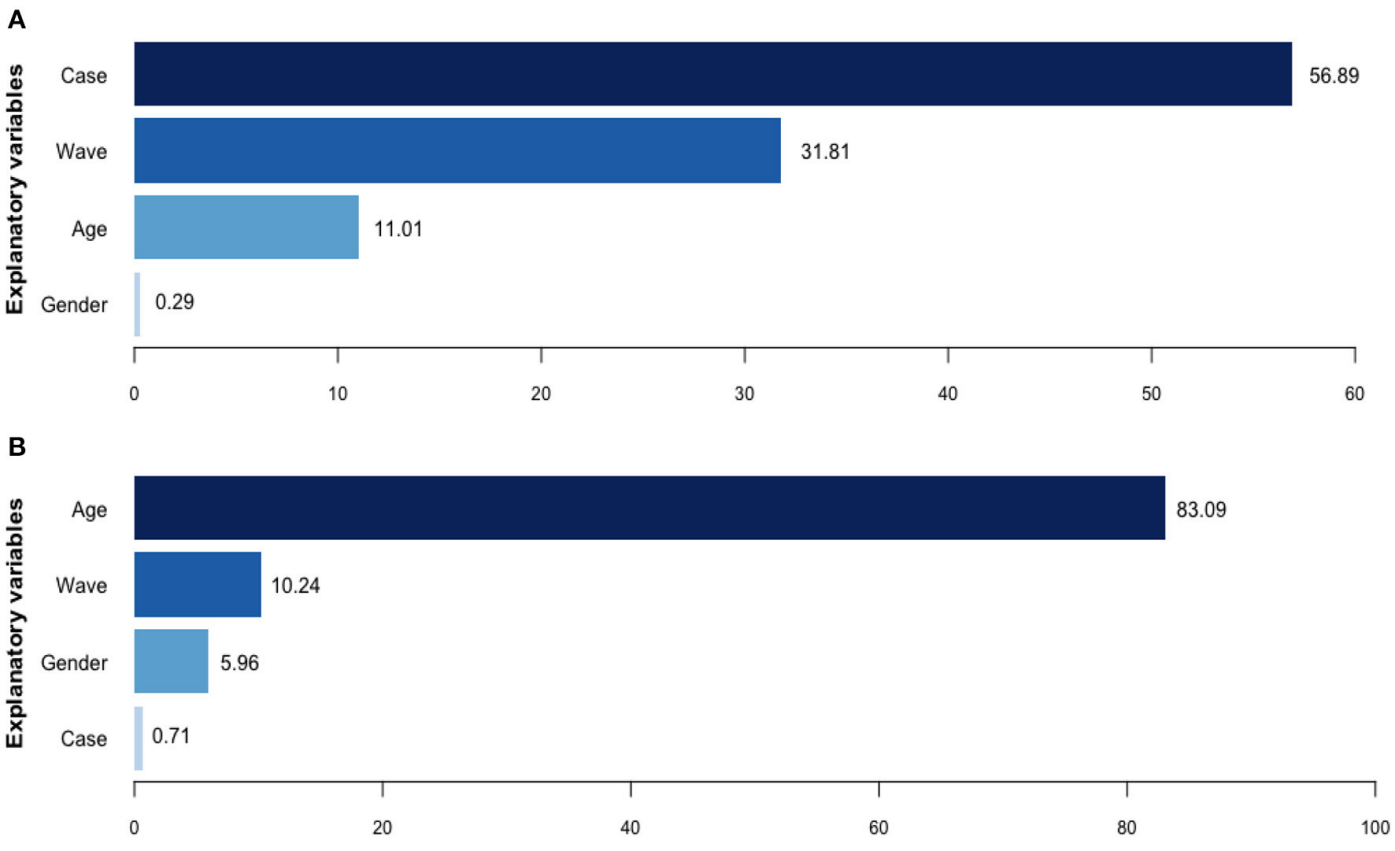
Relative contributions of potential explanatory variables for asymptomatic ratios and infection fatality ratios, by boosted regression tree model. **(A)** Relative contributions (%) for asymptomatic cases. **(B)** Relative contributions (%) for deceased cases.

Figure 1A shows the relative influence of each independent variable on the asymptomatic cases. Classification of imported or local cases contributed to over half of the relative importance (56.89%). The waves and patients' age had relative contributions of 31.81 and 11.01%, respectively. Gender only accounted for 0.29% of the importance of the BRT model.

Figure 1B shows the relative influence of each independent variable on the IFRs. Age was the dominating attributive factor for dead issues, contributing 83.09% of the BRT model's importance, followed by the wave dummy variables of COVID-19 (10.24%) and patients' gender (5.96%). Case classification contributed little to the deceased cases (0.71%).

**Figure 2:**
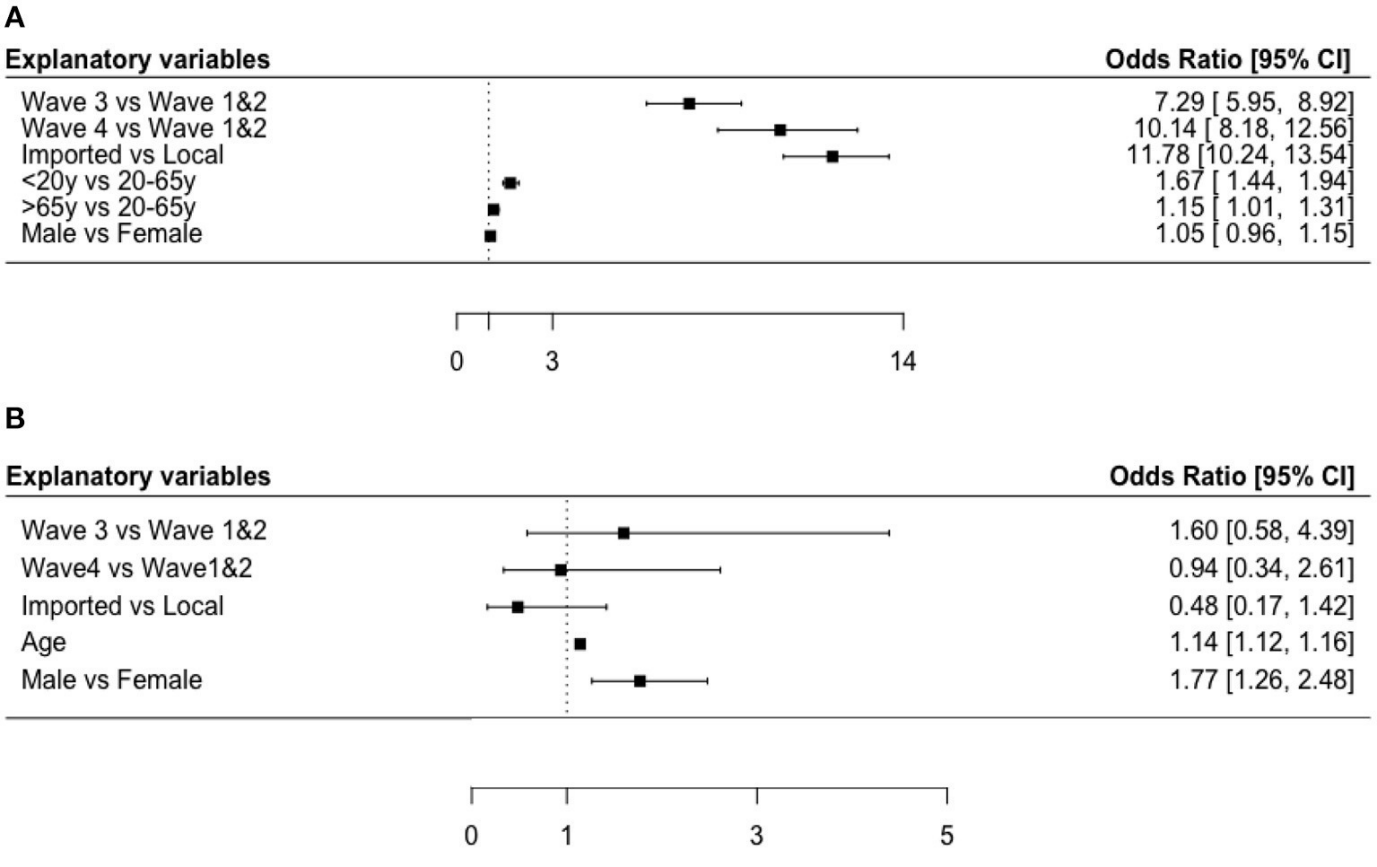
Forest plots of risk factors for asymptomatic cases and infection fatality ratios. **(A)** Odds ratio [95% CI] for asymptomatic cases in Hong Kong. **(B)** Odds ratio [95% CI] for deceased cases in Hong Kong.

Figure 2A shows that imported cases were more likely to be asymptomatic than local cases (OR:11.78, 95% CI: 10.24–13.54). Compared with cases in the first and second wave, the odds of asymptomatic cases were 7.29 times higher in the third wave (7.29, 5.95–8.92) and 10.14 times in the fourth wave (10.14, 8.18–12.56). We classified the age into three groups: <20 year, 20–65 years and >65 years to better explain the association between age and proportions of asymptomatic cases, since the odds ratio of asymptomatic cases for continuous variable of age was close to null, in spite of *P*-value <0.05. The results showed that patients aged <20 or >65 years were more likely to be asymptomatic cases than patients aged 20–65 years, after adjusting for other explanatory variables with an OR (95% CIs) of 1.67 (1.44–1.94) for under-20-year-old cases and 1.15 (1.01–1.31) for over-65-year-old cases. No significant association was reported between gender and the proportion of asymptomatic cases in the logistic regression.

Figure 2B also shows that people with higher age were at higher fatality risk with an OR (95% CI) of 1.14 (1.12–1.16). Besides, male patients had 1.77 times the odds of being deceased cases compared with females (OR: 1.77, 95% CI: 1.26–2.48). We also analyzed the association in the logistic regression by treating the variable as a continuous variable and found a significant association between the wave and IFR (AOR:0.69, 95% CI: 0.51–0.92). The results indicate that the IFR decreases in more recent waves probably due to the increased testing capability and healthcare capacity. No significant association was found between case classification and the proportion of deceased cases.

**Figure 3:**
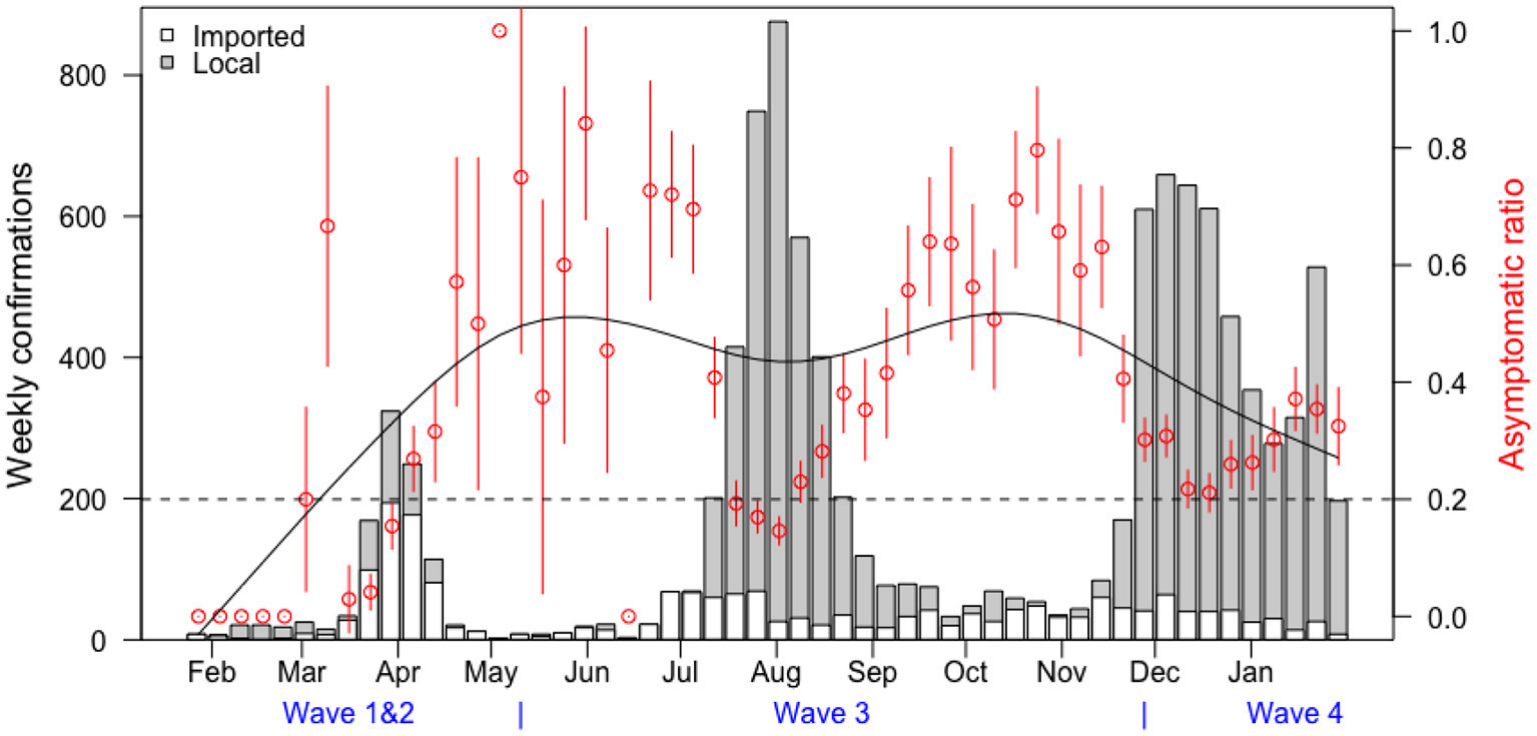
The time serial distribution of weekly confirmation and asymptomatic ratio.

Figure 3 visualizes the time serial distribution of weekly confirmation and asymptomatic ratio stratified by case classification and waves of COVID-19, the two most important explanatory variables for the asymptomatic cases. Imported cases were observed at the initial stage, while local cases dominated the latter two waves in Hong Kong. The proportion of asymptomatic cases fluctuated over time, always reaching the peak during remission periods.

**Figure 4:**
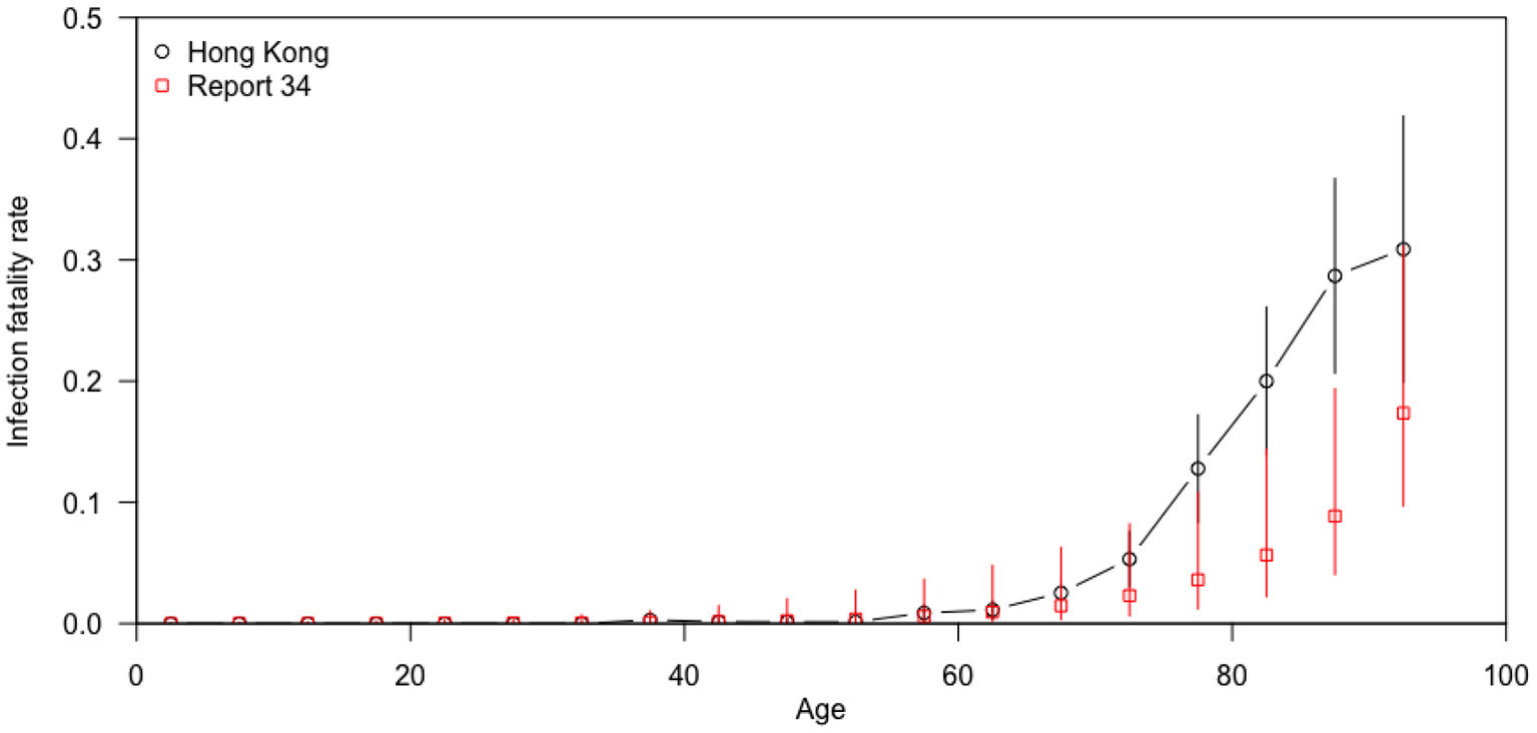
Infection fatality rates by age in Hong Kong and in other countries (14).

**Figure 5:**
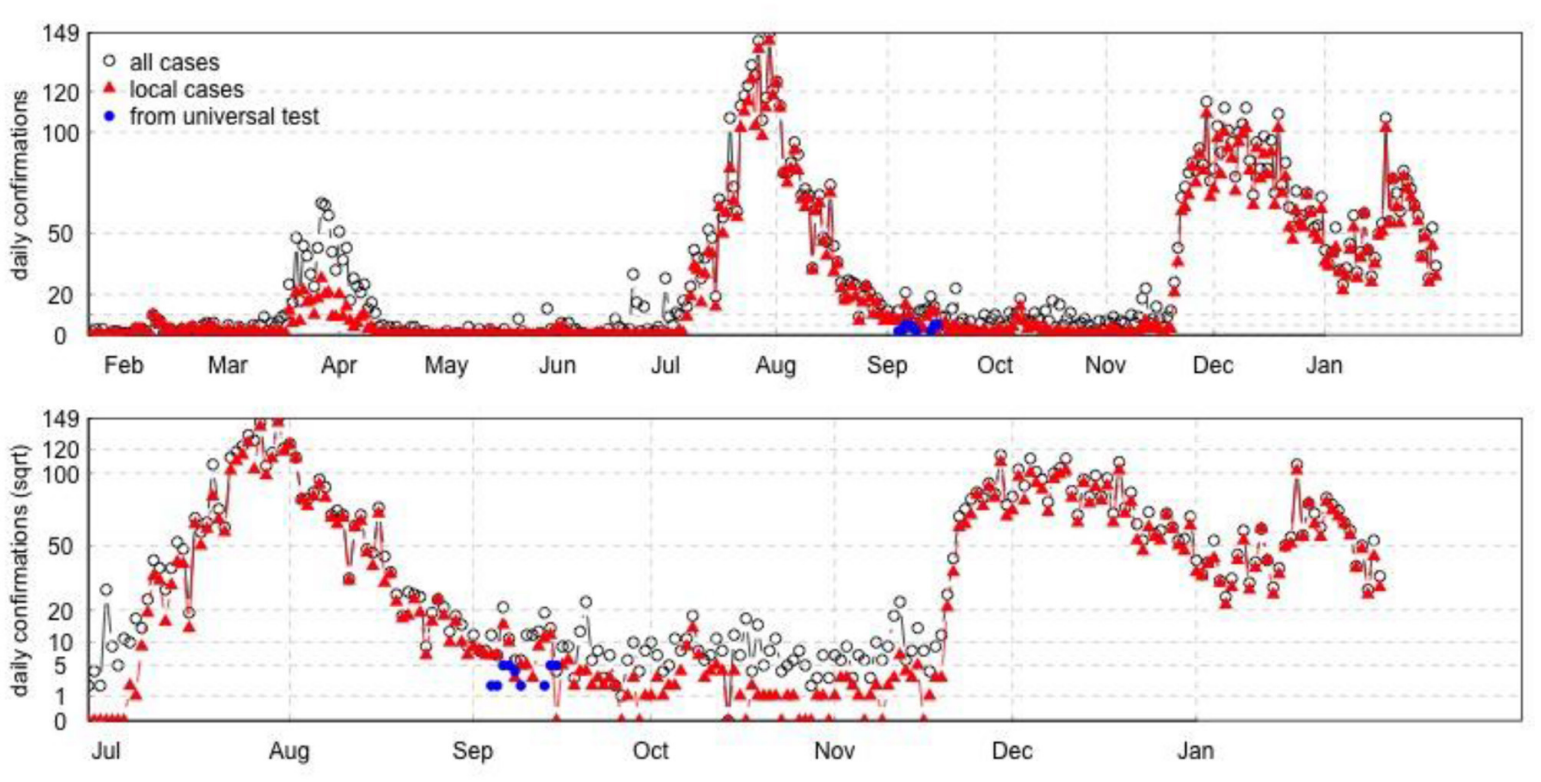
The time serial distribution of daily confirmed cases in Hong Kong.

Figure 4 compared the IFR by age in Hong Kong and other regions. The IFR increased dramatically after 60 years old, and such trend was more obvious in Hong Kong than in other regions. Figure 5 showed the waves of local COVID-19 transmission, which indicated the serial change of cases in Hong Kong.

## Discussion

This study is based on the number of the COVID-19 cases and the death cases collected in Hong Kong since the outbreak of the COVID-19 in early 2020. As of January 26th, 2021, the accumulated confirmed cases in Hong Kong was 10,223 (1,360/100,000,000) with a leading virus testing coverage of 901,940/100,000,000 around the world. With the non-pharmaceutical interventions (including border restrictions, social distancing, quarantine and isolation) in place (15), the COVID-19 local outbreaks in Hong Kong overall have not much overwhelmed the healthcare system and testing capability. This is crucial to ensure a reliable estimate of the asymptomatic case ratios and infection fatality rate compared with many other regions worldwide. According to previous reports, asymptomatic SARS-CoV-2 infection were more common in young people, and fatal cases were more common in older adults (16), which indicated the possible link between asymptomatic case ratio and IFRs. The estimated asymptomatic case ratios and IFRs should be able to represent the infection status among local residents in the latest two waves in Hong Kong given the testing capacity including the Universal Community Testing Programme launched in early September, 2020 which covered over 1.78 million residents in Hong Kong (9).

In our analysis, older age and male were significant risk factors for IFRs, which is consistent with previous studies (12, 17). A study combining age-specific mortality data from 45 countries with 22 seroprevalence surveys found that IFR was significantly higher in males than in females, and that IFR was positively associated with age among individuals aged 30 years or older (12). A meta-analysis of 27 studies showed an exponential relationship between age and IFR, starting with a very low rate in young adults and progressively increasing in the middle age (7). Consistently, the Imperial College COVID-19 response team in the UK identified 10 surveys from different countries (14) and showed a consistent pattern of age-specific IFR. Our study further compared the pattern with that reported by the Imperial College COVID-19 response team (14) and found the IFR for COVID-19 in Hong Kong was significantly higher, compared with other regions in the report, particularly in the elderly aged 65 years and older. The previous study has shown that the pattern of IFR by age is consistent across the globe among people aged below 65, while the pattern seemed to be more heterogeneous across settings in the elderly over 65 (12). Though we try to estimate the IFR as precisely as possible, it is also important to know that the overall IFR for COVID-19 might not be a fixed parameter because it depends on the age composition of the community and the extent to which the vulnerable population were exposed to SARS-CoV-2 (7).

Our study found the proportion of asymptomatic cases were higher in the third and fourth wave, possibly indicating an increase of test capacities in the latter waves. The higher proportion of asymptomatic among imported cases was reasonable because they should be “screened” before boarding and after arrival through taking temperature and the imported cases might have a higher social-economic status in general. In addition, the asymptomatic case ratios were higher during the interval of the local outbreaks and the government initiated a universal community testing campaign, shown in Figure 3, which might contribute to more precise IFR estimation, since the results from comprehensive tracing programs depend on the accuracy of the number of infected individuals, especially those asymptomatic cases (18).

Our study has several limitations. First, although the BRT model considered several factors, there were other potential factors that remained unadjusted. For example, the mortalities of COVID-19 patients with diabetes, cardiovascular diseases and hypertension were much higher than the general population (19). Unfortunately, individual data of pre-existing conditions were not available to us. Nevertheless, in this study we developed a modeling strategy which can be easily applied to the other regions/countries to assess a wider range of influencing factors. Second, although Hong Kong had a relatively higher testing rate compared to most of the other regions/countries, the possibility of underestimating the asymptomatic ratios cannot be ruled out. In the universal community testing campaign in Hong Kong, 1.78 million local residents (around 23.7% of the total local residents in Hong Kong) were tested, of whom only 42 were positive without any symptoms (9). Hence, we believe that the asymptomatic cases could have accounted for a very small proportion in Hong Kong local residents and should not greatly affect our conclusions.

Hong Kong has taken border restrictions with 14-day quarantine since the early outbreak in Mainland China following by the COVID-19 pandemic. The length of quarantine has been extended to 21 days after the mutant virus strain is reported at the end of December, 2020. The local community outbreaks have not yet overwhelmed the local capability of case track and record, which makes the COVID-19 cases recorded in this city among the most precise and representative over the world. In the regions suffering from high burden of COVID-19 cases, it is difficult to achieve such accurate estimation on case fatality. While in regions such as Singapore where the COVID-19 is under control with sporadic imported cases (20), it is hard to observe transmission in the local community which indicates that the case fatality might be not as representative as that in Hong Kong. With the non-pharmaceutical interventions in place, the sufficient testing capabilities and complete case recording infrastructure, the highly international city with 7.5 million residents has provided us with a unique data resource for understanding the disease profile amid the COVID-19 pandemic.

Although considerable investigation has been carried out to estimate the IFRs of COVID-19 in different settings, some have been challenged due to the non-representative samples such as the outbreak in the Diamond Princess Cruise, inappropriate statistical inference including falsely defaulting herd immunity in Brazil or duplicate use of data in meta-analysis (21). On the contrary, the estimates of asymptomatic ratio and case (infection)-fatality-rate in Hong Kong, China can be regarded as accurate given that (1) There are wide community transmission, thus local infections/cases are representative of the whole population; (2) There is extensive testing (under-reporting is less severe) so that the outbreak is under control; (3) The health system has not yet broken down. Considering few places elsewhere could satisfy these three conditions, the estimation of IFRs using surveillance data of COVID-19 in Hong Kong can be established as a standard among the comprehensive COVID-19 case tracing studies.

## Data Availability Statement

Publicly available datasets were analyzed in this study. This data can be found at: https://www.chp.gov.hk/files/pdf/local_situation_covid19_en.pdf.

## Author Contributions

JT and DH designed the study, carried out the statistical analysis, and wrote the manuscript. XZ, SM, and LY participated in data analysis and critically revised the manuscript. All authors contributed to the article and approved the submitted version.

## Conflict of Interest

DH was supported by an Alibaba (China)-Co. Ltd Collaborative Research Project. The remaining authors declare that the research was conducted in the absence of any commercial or financial relationships that could be construed as a potential conflict of interest.

## References

[1] Johns Hopkins University. COVID-19 Dashboard by the Center for Systems Science and Engineering (CSSE) at Johns Hopkins University (JHU) (2021). Available online at: https://gisanddata.maps.arcgis.com/apps/opsdashboard/index.html#/bda7594740fd40299423467b48e9ecf6 (accessed 8 March 2021).

[2] Wilder-Smith A Chiew CJ Lee VJ. Can we contain the COVID-19 outbreak with the same measures as for SARS? Lancet Infect Dis. (2020) 20:E102–7. 10.1016/S1473-3099(20)30129-8 32145768 PMC7102636

[3] Pollock AM Lancaster J. Asymptomatic transmission of covid-19. BMJ-Brit Med J. (2020) 371:m4851. 10.1136/bmj.m4851

[4] Mizumoto K Kagaya K Zarebski A Chowell G. Estimating the asymptomatic proportion of coronavirus disease 2019 (COVID-19) cases on board the diamond princess cruise ship, Yokohama, Japan, 2020. Euro Surveill. (2020) 25:2000180. 10.2807/1560-7917.ES.2020.25.10.2000180 32183930 PMC7078829

[5] Wells PM Doores KJ Couvreur S Nunez RM Seow J Graham C. Estimates of the rate of infection and asymptomatic COVID-19 disease in a population sample from SE England. J Infect. (2020) 81:931–6. 10.1016/j.jinf.2020.10.011 33068628 PMC7557299

[6] Xiao C Huang Z Wang J Zhao S Wong MCS Chong MKC. The Rate of Asymptomatic COVID-19 Infection: A Systematic Review and Meta-Analysis Including 12,713 Infections From 136 Studies. Modelling COVID-19 epidemics (2020).

[7] Levin AT Hanage WP Owusu-Boaitey N Cochran KB Walsh SP Meyerowitz-Katz G. Assessing the age specificity of infection fatality rates for COVID-19: systematic review, meta-analysis, and public policy implications. Eur J Epidemiol. (2020) 35:1123–38. 10.1007/s10654-020-00698-1 33289900 PMC7721859

[8] Havers FP Reed C Lim T Montgomery JM Klena JD Hall AJ. Seroprevalence of Antibodies to SARS-CoV-2 in 10 sites in the United States, March 23-May 12, 2020. JAMA Intern Med. (2020). 10.1101/2020.06.25.20140384 32692365 PMC12507447

[9] The Government of the Hong Kong Special Administrative region (2021). Available online at: https://www.info.gov.hk/gia/general/202009/15/P2020091500931.htm (accessed March 8, 2021).

[10] OT&P Healthcare. A Timeline of COVID-19 and OT&P Updates (2021). https://www.otandp.com/covid-19-timeline (accessed March 8, 2021).

[11] Tao J Zhang X Zhang X Zhao S Yang L He D. The time serial distribution and influencing factors of asymptomatic COVID-19 cases in Hong Kong. One Health. (2020) 10:100166. 10.1016/j.onehlt.2020.100166 32904481 PMC7455807

[12] O'Driscoll M Ribeiro Dos Santos G Wang L Cummings DAT Azman AS Paireau J. Age-specific mortality and immunity patterns of SARS-CoV-2. Nature. (2020) 590:140–5. 10.1038/s41586-020-2918-0 33137809

[13] Wong J Abdul Aziz ABZ Chaw L Mahamud A Griffith MM Lo YR. High proportion of asymptomatic and presymptomatic COVID-19 infections in air passengers to Brunei. J Travel Med. (2020) 27:taaa066. 10.1093/jtm/taaa066 32365178 PMC7239182

[14] Brazeau NF Verity R Jenks S Fu H Whittaker C Winskill P. COVID-19 Infection Fatality Ratio: Estimates from Seroprevalence. London: Imperial College London (2020).

[15] Cowling BJ Ali ST Ng TWY Tsang TK Li JCM Fong MW. Impact assessment of non-pharmaceutical interventions against coronavirus disease 2019 and influenza in Hong Kong: an observational study. Lancet Public Health. (2020) 5:e279–88. 10.1016/S2468-2667(20)30090-6 32311320 PMC7164922

[16] Ren R Zhang Y Li Q McGoogan JM Feng Z Gao GF. Asymptomatic SARS-CoV-2 infections among persons entering China from April 16 to October 12, 2020. JAMA. (2021) 325:489–92. 10.1001/jama.2020.23942 33528529 PMC7856538

[17] Mallapaty S. The coronavirus is most deadly if you are old and male. Nature. (2020) 585:16–17. 10.1038/d41586-020-02483-2 32860026

[18] Vermund SH Pitzer VE. Asymptomatic transmission and the infection fatality risk for COVID-19: implications for school reopening. Clin Infect Dis. (2020) 72:1493–6. 10.1093/cid/ciaa855 32584967 PMC7337644

[19] Riddle MC Buse JB Franks PW Knowler WC Ratner RE Selvin E. COVID-19 in people with diabetes: urgently needed lessons from early reports. Diabetes Care. (2020) 43:1378–81. 10.2337/dci20-0024 32409505 PMC7305002

[20] Ngiam JN Chew N Tham SM Beh DL Lim ZY Li TYW. Demographic shift in COVID-19 patients in Singapore from an aged, at-risk population to young migrant workers with reduced risk of severe disease. Int J Infect Dis. (2020) 103:329–35. 10.1016/j.ijid.2020.11.157 33220440 PMC7674985

[21] Shen C VanGennep D Siegenfeld AF Bar-Yam Y. Unraveling the flaws of estimates of the infection fatality rate for COVID-19. J Travel Med. (2021) 28:taaa239. 10.1093/jtm/taaa239 33398337 PMC7798978

